# Septic rupture of the ascending aorta after aortocoronary bypass surgery

**DOI:** 10.1186/1749-8090-3-64

**Published:** 2008-12-16

**Authors:** Christof M Sommer, Tobias Heye, Ulrike Stampfl, Ursula Tochtermann, Boris A Radeleff, Hans U Kauczor, Goetz M Richter

**Affiliations:** 1 Department of Diagnostic and Interventional Radiology, University Hospital Heidelberg, Heidelberg, Germany; 2 Department of Cardiac Surgery, University Hospital Heidelberg, Heidelberg, Germany

## Abstract

We describe an exceptional case of non-fatal septic rupture of the ascending aorta in a patient with sternal dehiscence, deep sternal wound infection (DSWI) and pleural empyema after aortocoronary bypass surgery. Routine follow-up computed tomography (CT) detected a mediastinal pseudoaneurysm originating from the ascending aorta. Thereby, massive and irregular sternal bone defects and contrast-enhancing mediastinal soft tissue suggest osteomyelitis and highly-active and aggressive DSWI as initial triggers. Urgent thoracotomy 1 day later included ascending aorta reconstruction, total sternum resection and broad wound debridement. Follow-up CT 1 year later showed a regular postoperative result in a fully recovered patient.

## Background

Sternal dehiscence is a frequent complication after aortocoronary bypass surgery with an incidence estimated at 1% to 6% in a normal population [1]. When complicated by sternum osteomyelitis and DSWI, prolonged hospitalization and increased cost of care, morbidity and mortality as high as 20% is reported [2]. Broken wire fixations and displaced bone fragments with non-physiological sternal movement seem to be essential in its pathogenesis [3]. Obesity, diabetes mellitus, chronic obstructive pulmonary disease and previous sternotomy make up the major risk factors [4]. Despite antibiotic therapy and aggressive surgical interventions, the prognosis is serious [5]. In this case report, we describe a patient developing non-fatal septic rupture of the ascending aorta with mediastinal pseudoaneurysm triggered by sternum osteomyelitis and extensive DSWI after aortocoronary bypass surgery.

## Case presentation

**Figure 1:**
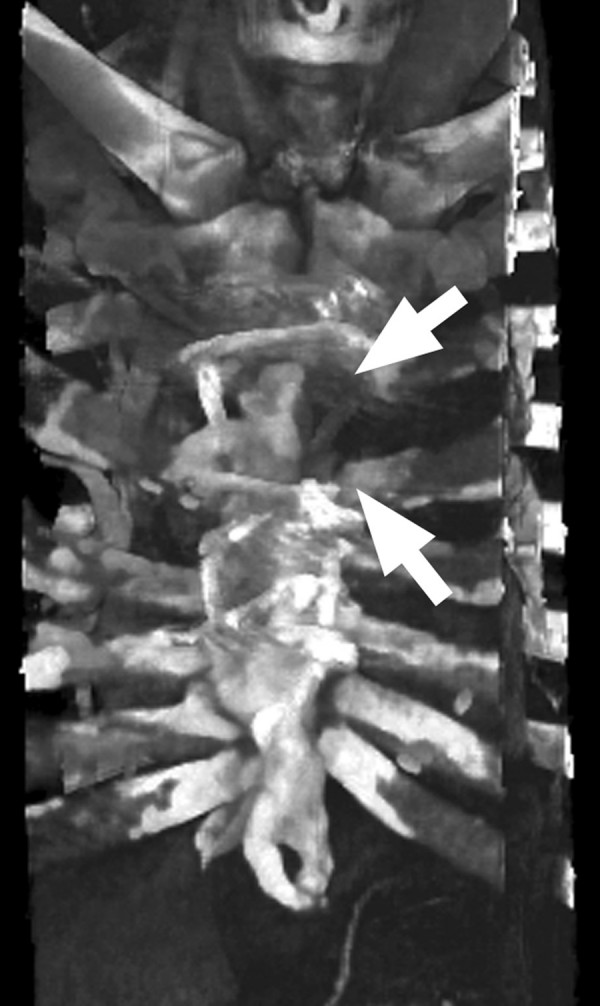
**Sternum osteomyelitis**. CT 3D-reconstruction shows massive sternal bone defects, inhomogenous bone matrix and irregular sternotomy edges (white arrows) as evidence for long-lasting chronic osteomyelitis

**Figure 2:**
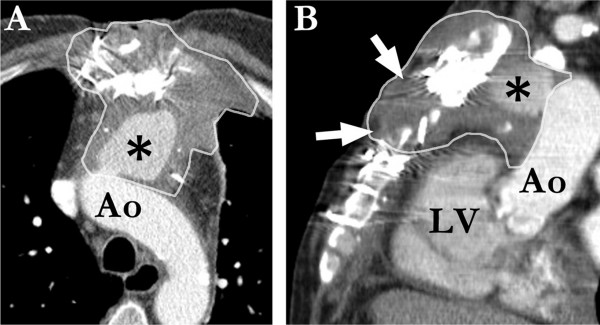
**DSWI with pseudoaneurysm**. CT axial (A) and sagittal (B) reconstructions show clearly contrast-enhancing soft tissue in the upper and anterior mediastinum (areas encirceled by white lines); ascending aorta (Ao), pseudoaneurysm (asterisk), left ventricle (LV) and massive sternal bone defects (white arrows).

A 65-year-old male white was referred to our department for routine follow-up CT for sternal dehiscence, DSWI and pleural empyema developing after aortocoronary bypass operation 9 months before. His history included congenital portal vein stricture with recurrent ascites, acute myocardial infarction 4 months ago, systemic hypertension, hypercholesteremia, renal insufficiency, penicillin allergy and methicillin-resistant staphylococcus aureus in the blood culture. Medication consisted of spironolactone, metoprolol succinate, furosemide, ramipril, acetylsalicylate, zinc aspartate and zolpidem tartrate. Physical examination was carried out in the Department of Cardiac Surgery right before the CT and revealed pressure-like pectoral pain and non-physiological sternal movement. Laboratory showed elevated c-reactive protein (58 mg/l), ferritin (1450 μg/l) and gamma-glutamyl transferase (77 U/l), whereas choline esterase (3110 U/l) and hemoglobin (12.5 g/dl) were reduced. Electrocardiogram and tropoine T were unremarkable. CT detected massive sternal bone defects, inhomogenous bone matrix and irregular sternotomy edges as evidence for long-lasting chronic osteomyelitis (Figure 1). Clearly contrast-enhancing soft tissue in the upper and anterior mediastinum documeted highly-active and aggressive DSWI (Figure 2A, B). An oval-shaped massive contrast-enhancing retrosternal mass directly within the DSWI with narrow connection to the ascending aorta indicated a mediastinal pseudoaneurysm (Figure 2). Because hyperacute aortic rupture was unlikely due to postoperative mediastinal scarring, the patient underwent urgent thoracotomy 1 day later. Ascending aorta reconstruction with insertion of a pericardial patch, total sternum resection, broad wound debridement inclusive of soft tissue reconstruction with a pectoralis plastic was carried out. Intraoperative mediastinal bacterial smear tests verified existence of methicillin-resistant staphylococcus aureus. Postoperative CT 2 days later demonstrated a proper thoracic aorta. 12 days later, the patient was transferred from intensive care unit to ward. Another 8 days later, he was admitted to a peripher hospital in good clinical condition. Outpatient routine follow-up CT 1 year later documented a regular postoperative result in a fully recovered patient.

## Conclusion

Midline sternotomy is by far the most common surgical approach in cardiac surgery. One disadvantage of this technique is the relatively frequent occurence of sternal dehiscence with instability [1]. It is described as non-physiological sternal movement after disruption of wire fixations [3]. Because of its decreased blood supply, especially the lower sternal third is affected. Moreover, utilization of the internal mammary artery for coronary artery bypass grafting leads to additional and acute reduction of the sternal blood supply [6]. When this situation is complicated by infection, which occurs especially in patients with immunosuppression, sternum osteomyelitis and DSWI might develop as devastating complications despite strict sterility and antibiotic prophylaxis [7]. In case of aggressive mediastinal propagation, cardiac suture lines might be eroded with septic shock or hemorrhage as fatal complications [7]. It is well known, that continuous bacterial spread from or within a soft tissue compartment might cause vascular infection characterized as bacterial aortitis, infected aorta, ruptured mycotic aneurysm or spontaneous non-aneurysmal suppurative vascular rupture [8]. Sugawara et al. described a ruptured abdominal aorta secondary to klebsiella pneumonia psoas muscle abscess [9]. The typical mechanism of aortic rupture, which is expansion of an atherosclerotic aneurysm, is virtually impossible in our patient. Neither the short period of only 9 months nor pseudoaneurysm configuration directly within the DSWI support this. Much more probable is chronic septic vascular erosion resulting in aortic rupture with pseudoaneurysm formation. Aggressive surgical interventions with broad wound debridement, plastic reconstruction, bone removal and mediastinal irrigation with antiseptic solutions in combination with intravenous antibiotic therapy seem to be the only chance to control extensive sternal osteomyelitis with DSWI and to prevent even worse complications [5]. Accomplished in our patient, admission to a peripher hospital was possible in good clinical condition 22 days later and outpatient routine follow-up CT 1 year later documented a regular postoperative result in a fully recovered patient.

## Consent

Written informed consent was obtained from the patient for publication of this case report and accompanying images. A copy of the written consent is available for review by the Editor-in-Chief of this journal.

## Competing interests

The authors declare that they have no competing interests.

## Authors' contributions

CMS conceived the study. TH prepared the figures. CMS and TH performed the CT-examination. CMS and US did the background literature search and drafted the manuscript. UT was the cardiac surgeon. BAR participated in study design and coordination. HUK and GMR were involved in the conception of the study and the critical review of the intellectual content of the manuscript. All authors have read and approved the final manuscript.
